# The Dental Protective Association of the United States

**Published:** 1893-06

**Authors:** J. N. Crouse

**Affiliations:** Chairman; Chicago


### THE DENTAL PROTECTIVE ASSOCIATION OF THE UNITED STATES.

To the Editor:

Sir,—We herewith send you an account of the Dental Protective Association, showing what we have done, what we are doing, and what we expect to do for each one who belongs to the Association.

*First.*—We have freed the dental profession from annoyance or from paying royalty to the International Tooth-Crown Company for the past four years. We have also stopped twelve or thirteen other patent companies from collecting and canvassing for royalties.

We have thus saved the dental profession a million dollars a year; for, without the protection given by this Association, every practitioner in the United States would have been compelled to pay royalty on some of the numerous patents owned by these companies, and there are in the United States between sixteen thousand and eighteen thousand dentists. It has saved each practitioner not less than one hundred dollars annually for the past four years. So much for a very brief summary of what we have done.

*Second.*—We are defending, at present writing, suits brought against our members in Indiana, Michigan, Ohio, New York, Connecticut, and Colorado. We are having one important test suit with the International Tooth-Crown Company on the Low Bridge patent. This suit has been in process of preparation for over two years, and has required a large expenditure of money, time, and energy.

Members of the Dental Protective Association have been sued during the last year for an infringement of letters-patent granted C. H. Land on what is known as "Inlays."

The chief part of this patent consists of the taking of an impression by burnishing either platinum or gold into and accurately around the edges of the cavity, thus forming a matrix, and then filling this matrix with porcelain or some substance which is melted into it. The plug thus made is secured by cement.

I should be much pleased to have a detailed statement from all members of the profession who practised this method prior to 1886.

Again, we examine *all* patents in which our profession is interested, and constantly give information to members of the Association on patent claims, thus saving them from paying royalty on unjust patents.

We are now investigating the various remedies and methods for painless dentistry, and we shall be prepared to give to all our members the secrets and analysis of the remedies by the time this circular reaches them.

Thus far in the history of the profession a dentist's expenses have necessarily been out of all proportion to his income; but a new era is before us, and soon, we trust, it will not be necessary for a dentist to wait five years before he can save ten dollars to join the Dental Protective Association.

The work of the Dental Protective Association of the United States has been so entirely successful, and has been carried on with such *apparent* ease, that the profession at large, and even the members, I am led to think, but faintly realize the amount of time and labor it has involved during the five years since its organization.

The defence of the suits brought by the International Tooth-Crown Company alone has necessitated taking the testimony of nearly one hundred witnesses in all parts of the country, each of whom has been secured by the chairman, who has also been present at their examination. Besides this, the vast numbers of letters written, the thousands of miles travelled, the weeks of time given in attending public meetings, examining patents, securing witnesses, defending suits of members, etc., etc.,—all this has been given freely and cheerfully, but, let it be distinctly understood, without one dollar of compensation; on the contrary, in the beginning of the organization the chairman not infrequently used his own funds in defraying expenses.

This explanation is made in order that no one may hesitate to join through fear that his membership fee may be spent in paying salaried officers or benefiting the management.

BENEFITS OF THE DENTAL PROTECTIVE ASSOCIATION.

It saves the profession millions of dollars.

It saves its members time and annoyance from the visits of patent claimants.

It saves its members the time, the worry, and the expense of defending their own suits.

It wins their suits when they, individually, would fail.

It examines all dental patents and gives its members the benefit.

It examines new remedies and methods and gives its members the process *free.*

It furnishes its members (or will soon) materials at about one-half the usual prices.

It furnishes membership in the Association for *ten* dollars.

It has no annual dues or assessments.

*Does it pay* to be a member of the Dental Protective Association?

All communications should be sent to

J. N. CROUSE,
*Chairman.*

CHICAGO, April 27, 1893.

